# Entrepreneurial Passion and Venture Capitalists’ Willingness to Invest: The Role of Relational Capital

**DOI:** 10.3389/fpsyg.2021.728589

**Published:** 2021-11-11

**Authors:** Hongtao Yang, Hangyu Shi, Yenchun Jim Wu, Lei Zhang, Shuting Xie

**Affiliations:** ^1^School of Business Administration, Huaqiao University, Quanzhou, China; ^2^Graduate Institute of Global Business and Strategy, National Taiwan Normal University, Taipei, Taiwan; ^3^Leisure & Recreation Administration Department, Ming Chuan University, Taipei, Taiwan

**Keywords:** entrepreneurial passion, venture capitalists, willingness to invest, relational capital, signaling theory

## Abstract

Venture capital investment has serious conflicts of interest and information asymmetry. Venture capitalists often make investment decisions on the basis of the passion of entrepreneurs, including enthusiasm and preparedness, in the process of interacting with them. Most of the previous research on relational capital have focused on the cooperative relationship between suppliers and buyers. However, the role of relational capital in the process of partnership between venture capitalists and entrepreneurs has not been revealed. On the basis of signaling theory, we explore the relationship between entrepreneurial passion and venture capitalists’ willingness to invest. We also examine the mediating and moderating roles of relational capital. This study takes 79 projects between venture capitalists and entrepreneurs as samples for empirical analysis to verify our hypothesis. Results show that entrepreneurs’ enthusiasm and preparedness have a significant positive impact on venture capitalists’ willingness to invest. Relational capital plays a mediating role between entrepreneurial passion and venture capitalists’ willingness to invest. Relational capital positively moderates the relationship between preparedness and venture capitalists’ willingness to invest but has no moderating effect between enthusiasm and venture capitalists’ willingness to invest. Results deepen the understanding of the relationship between entrepreneurs’ passion and venture capitalists’ willingness to invest, which has guiding significance for venture capital practice in China.

## Introduction

In the early stages, the biggest problem usually faced by a startup is limited resources, and 80% of enterprises regard financing constraints as one of the main obstacles to their development (Claessens and Tzioumis, 2006). To grow their business, entrepreneurs need to continuously seek external financial support (Amit et al., 1990), which includes raising funds in the form of venture capital (Jeng and Wells, 2000). However, the process of securing investment in the early stages is difficult, because venture capitalists are cautious about investing in entrepreneurs. Many scholars have studied the reasons for this based on stakeholder perspective (Plummer et al., 2016; Bi et al., 2017) and risk perspective (Löher, 2017; Vismara, 2018). As a result, how entrepreneurs can gain venture capital from venture capitalists in the early stage of business has become a key concern for academia and practitioners.

New venture financing is an ongoing process (Ko and McKelvie, 2018). Most of the existing studies focus on the initial selection or screening stage, which is the stage where most deals fail because venture capitalists are unable to make rational decisions on a short period of time relying on limited information (Cardon et al., 2017). Scholars have established that venture capitalists’ willingness to invest is not only related to the objective factors, such as entrepreneurs’ human capital (Collewaert and Manigart, 2016; Murnieks et al., 2016), and entrepreneurial team experience capabilities (Harrison et al., 2015), but also to entrepreneurs’ entrepreneurial passion (Cardon et al., 2017), coachability (Ciuchta et al., 2018), and other subjective factors. Furthermore, entrepreneurial passion is an important factor in how entrepreneurs can gain the favor of venture capitalists in the short process. Entrepreneurial passion has been shown to drive entrepreneurs to pursue their goals tenaciously and to compel stakeholders to support venture capital. Chen et al. (2009) assert that passion plays an important role for investors in making decisions. Cardon et al. (2009) argue that venture capitalists consider the passion exhibited by entrepreneurs an important factor in their investment decisions. Although studies have been conducted to reveal the influence of entrepreneurial passion on the investment behavior of venture capitalists, scholars have defined the definition of entrepreneurial passion and classified its dimensions with different criteria. For example, passion in the individual trait perspective is an innate trait of the entrepreneur himself or herself (e.g., Baum and Locke, 2004), whereas passion in the emotion perspective is a conscious, ongoing, accessible, and positive emotional experience of the entrepreneur (e.g., Cardon et al., 2009, 2017). Whether the trait perspective of passion affects investors’ willingness to invest or the emotional perspective of passion affects investors’ willingness to invest more will need to be further explored in future research. The impact of entrepreneurial passion divided by different dimensions on venture capitalists’ investment decision-making behavior is bound to be different. Therefore, this study further examines the effect of entrepreneurial passion on venture capitalists’ willingness to invest.

In facilitating a partnership, entrepreneurs face the challenge of convincing venture capitalists to invest in their business, whereas venture capitalists seek to overcome information asymmetry and minimize investment risks. Therefore, startups must overcome the skepticism of venture capitalists toward entrepreneurs (Ko and McKelvie, 2018). In this aspect, signal theory can explain this problem thoroughly. Signaling theory suggests that in a scenario of information asymmetry, the party lacking information will infer the true information from signals (Spence, 2002). As Huang and Knight (2017) point out, entrepreneurs can send informational signals and interpersonal signals to investors, who weigh them before entering into a relationship. Investors can consider whether an entrepreneur is ready to run a business according to how well the entrepreneur thinks about his or her business. Substantial research has been conducted on how signals influence investment decision behavior, yet only a little research is found on the relationship between signals and investment decisions in the early stages of venture development (Ko and McKelvie, 2018). How entrepreneurial passion affects venture investment decisions is even more exploratory.

The effective transmission of a signal is not only related to the content of the signal, but also to how the receiver perceives and interprets the signal (Ciuchta et al., 2018). Wang (2016) argue that when convincing investors to invest, entrepreneurs can hide information, which, in turn, affects venture capitalists’ overall assessment of further investments. Thus, although investors receive positive signals from entrepreneurs, they are concerned with the opportunistic risk of entrepreneurs, thus making cautious decisions (Cardon et al., 2017). Relational capital is a long-term asset that can be invested in other resources and can predict future benefit flows (Adler and Kwon, 2002). Our study argues that relational capital can effectively alleviate this dilemma. We consider relational social capital because studies have found that relational capital can reduce this concern by reducing expectations of opportunistic behavior, thereby increasing trust and reducing transaction costs (Dyer and Singh, 1998). Our study argues that the relational capital perceived by venture capitalists during their interactions with entrepreneurs can reduce their vigilance in signaling entrepreneurial passion to entrepreneurs. Therefore, the paper reveals the mechanism of the role of the entrepreneurial passion of entrepreneurs on venture capitalists’ willingness to invest based on signaling theory and introduces relational capital in the process to reveal how entrepreneurial passion affects venture capitalists’ willingness to make decisions. Furthermore, relational capital can be measured through the constructs of trustworthiness and trust. When relationships are built on the basis of trust, individuals also choose to trust a person or a group of people according to trustworthiness (McAllister, 1995). Interpersonal relationships based on high levels of trust and emotion can effectively reduce deception and opportunistic behavior (Abdullah et al., 2019). For example, Blonska et al. (2013) showed that relational capital is the “bridge” between supplier development and relationship benefits. When suppliers and partners trust each other, they are more inclined to cooperate. Entrepreneurs with high relational capital are likely to convey more credibility and trust to venture capitalists, which makes the entrepreneurial passion conveyed by entrepreneurs easier to be interpreted by venture capitalists in the signaling process, which in turn generates a willingness to make investment decisions. Therefore, we further examine the moderating role of relational capital in entrepreneurial passion and venture capitalists’ willingness to invest.

In summary, our study is based on signaling theory and introduces relational capital as a mediating and moderating variable to explore the mechanism of entrepreneurial passion’s influence on venture capitalists’ willingness to invest. We aim to make three substantive theoretical and practical contributions. Firstly, the positive role of entrepreneurial passion has been a hot topic in the field of venture capital, but the role of entrepreneurial passion in influencing early stage development venture capital decisions has not been adequately discussed in the previous literature, especially the lack of empirical studies on its mechanisms. Therefore, the current study examines the mechanism of the influence of entrepreneurial passion for venture capitalists’ willingness to invest based on signaling theory. Our findings not only enrich the existing research on the investigation into signaling theory but also provide theoretical and empirical support for explaining the role of entrepreneurial passion for venture capitalists’ willingness to invest an investment. Second, we introduce the concept of relational capital to the stage before investors and entrepreneurs form a partnership, and defines the boundaries role of entrepreneurs releasing signals of entrepreneurial passion to influence the decisions of venture capitalists through the perception and interpretation of signals by venture capitalists. Most previous studies on relational capital have focused on the supplier-buyer partnership (Blonska et al., 2013). Our study extends a broader understanding and application of relational capital, thus making a theoretical contribution to social capital theory. Finally, we assess the mechanism of entrepreneurial passion’s influence on venture capitalists by observing the true interaction process between entrepreneurs and venture capitalists. We rely on the observational interaction method to collect data that more objectively and truly reflects the investment decision-making process of venture capitalists, breaking through the traditional questionnaire collection method, and getting rid of relying on the recall answer of venture capitalists and investors. This approach has an important role in understanding venture capitalists’ willingness to invest.

## Theoretical Overview and Hypotheses

### Entrepreneurial Passion and Venture Capitalists’ Willingness to Invest

Research on passion is emerging. Such passion has been shown to drive entrepreneurs to pursue their goals tenaciously and to compel stakeholders to support venture capital. Scholars have defined entrepreneurial passion primarily from an emotional perspective and a motivational perspective. Scholars consider entrepreneurial passion as a conscious, sustained, accessible, and positive emotional experience. Entrepreneurs who are passionate about entrepreneurship are often motivated to understand their entrepreneurial identity better and to behave accordingly (Vallerand et al., 2003; Cardon et al., 2009). Chen et al. (2009) view passion as a motivation that stimulates the thoughts and behaviors of entrepreneurs. In this study, the concept of entrepreneurial passion uses Chen et al. (2009) definition of entrepreneurial passion as a strong emotional state in which entrepreneurs have high personal values in terms of cognitive and behavioral performance, where the cognitive aspect is reflected as enthusiasm and the behavioral aspect is reflected as preparedness.

Passion in the entrepreneurship literature emphasizes emotions, especially positive emotions (Chen et al., 2009), which Baum and Locke (2004) defined as “love of work.” Consistent with Chen et al.’s (2009) definition of enthusiasm, we believe that enthusiastic entrepreneurs not only have positive emotions, but their minds are always active, such that they constantly think about what they can do for their business. Previous research on entrepreneurial enthusiasm is scant, and some scholars have studied the assimilation of the concepts of enthusiasm and passion. Entrepreneurial enthusiasm plays an important role in investors’ investment decisions (Cardon et al., 2017). Entrepreneurs who exhibit high enthusiasm increase investor interest in a business by 26% relative to those who exhibit low enthusiasm (Shane et al., 2020). Owing to the nature of the entrepreneur-venture capitalist relationship, signals play an important role in their early interactions. Signal theory is concerned with reducing asymmetric information between parties (Spence, 2002) and suggests that actors consciously and voluntarily attend to available signals to reduce perceived uncertainty (Spence, 1974). Enthusiasm is the primary emotional signal of an entrepreneur; it conveys his basic emotions toward the business (Cardon et al., 2017). First, empirical evidence suggests that enthusiasm provides entrepreneurs with work-related self-efficacy (Suvittawat, 2019), that is, the belief that they have the personal resources and willpower to succeed, and the positive belief that they can achieve the desired outcome. Individuals with high self-efficacy will be more active on a given task for a longer period, work harder in difficulties, set and accept higher goals, and have better plans and strategies for task completion (Shane et al., 2003). Thus, showing enthusiasm is a way for an entrepreneur to communicate to investors that he has high ability and persistence for entrepreneurial activity. Second, enthusiasm can make entrepreneurs more persuasive (Baron, 2008). Cardon (2008) believed that experienced deep emotion is more likely to be expressed. Entrepreneurs transmit their enthusiasm for entrepreneurial activities to venture capitalists by expressing their positive emotions through body and verbal language and facial expressions during their interactions with them (Chen et al., 2009). Previous research on entrepreneurship has suggested that emotional expression plays an important role and that positive emotions are persuasive (Hatfield et al., 1993; Rucker and Petty, 2006). Thus, entrepreneurs showing enthusiasm can be more persuasive.

Signal theory suggests that signals provide information aimed at changing the receiver’s understanding of future states (Busenitz et al., 2005). The effectiveness of signals also depends on the receiver’s ability to detect them and the importance attached to them (Connelly et al., 2011). Therefore, signals have a more important dimension to investment decisions than other factors: they need to be perceived and interpreted by investors (Courtney et al., 2017). The behavior of investors in interpreting signals may be influenced by the personal traits of investors. Mitteness et al. (2012) explored the relationship between individual traits of venture capitalists on the perceived passion of entrepreneurs and the assessment of investment potential. Part of the research appears in the behavioral finance literature, where scholars view investors as not entirely rational and understand decisions as potentially influenced by overconfidence, over-optimism, and other irrational behaviors (Linder and Sperber, 2020). Thus, if investors have a behavioral bias of overconfidence in the investment decision process, then they will tend to overestimate their information processing ability and be overconfident in their interpretation of signals, thus creating an over-reliance on signals in their decision making. In addition, enthusiasm can more easily induce overconfident behavior in venture capitalists. Emotional contagion exists in communication between individuals or between members of a group (Hatfield et al., 1993). When investors are in a heightened emotional state after being infected with enthusiastic emotions from entrepreneurs, their behavioral decisions tend to select and process information consistent with that emotion (Bower, 1981). Scholars have argued that when investors are in a high or optimistic mood, they tend to predict project prospects optimistically and increase their trust in the accuracy of the information they obtain. In summary, entrepreneurial enthusiasm is more persuasive than other signals to venture capitalists and increases the perceived value of a signal to venture capitalists.

Entrepreneurial preparedness is another critical signal that influences venture capitalists’ willingness to invest (Chen et al., 2009). Chen defined preparedness as the extent to which an entrepreneur thinks and thinks about particular aspects of his or her business. Startups can obtain external resources in the early stages by signaling to venture capitalists that they have the potential to return a significant amount on investment. However, at the early stages of growth where failure rates are high, the future of startups is largely uncertain (Ko and McKelvie, 2018). Startups lack sufficient evidence that their value proposition is viable and that their management teams are capable of managing their companies (Ostgaard and Birley, 1996). The perspective of the interpretation of signals by venture capitalists shows that the signals delivered by entrepreneurs lack credibility. Therefore, scholars have argued that entrepreneurs package information about the firm’s existing resource endowment into an appealing format that can increase the veracity of the readiness signals sent by venture capitalists to entrepreneurs (Cardon et al., 2017). First, entrepreneurs that have given a great deal of thought to their venture and the associated business environment can provide a coherent, thoughtful, and detailed story about their venture and its future. This packaged information is more memorable, carries more weight, and is more likely to influence the behavior of others, including venture capitalists (Swap et al., 2001; O’Connor, 2002; Nagy et al., 2012). According to signal theory, the more vivid and informative the story the entrepreneur tells, the more likely the venture capitalist is to determine whether this information meets their evaluation criteria. Second, the interaction between entrepreneurs and venture capitalists is a process of persuasion. On the one hand, entrepreneurs can convey plans and ideas for the future development of their company in the process of convincing investors. On the other hand, investors can consider whether the entrepreneur is prepared to run the enterprise according to the degree of thinking of the entrepreneur, or may turn out to be a risky partner who may give up halfway on a whim, which is considered more important by investors than the entrepreneur’s enthusiasm (Cardon et al., 2009). Moreover, in the practice of entrepreneurship, scholars argue that venture capitalists usually invest in people, not projects, in the early stages of venture capital (Sahlman, 1997). The expertise of the messenger affects the degree to which the information is received by the receiver (Ohanian, 1990). Entrepreneurs’ clarity in the presentation process regarding the funded and soon-to-be-funded arguments reflects their expertise to some extent. This expertise in a sense represents the entrepreneur’s prior knowledge and experience, which has a positive effect on venture capitalists’ willingness to invest. Finally, the failure tolerance of venture capitalists also influences their interpretation of preparedness signals. Investors with a high tolerance for failure regard the long-term potential value of the firm (Tian and Wang, 2014). Previous studies indicate that transparency of information can increase tolerance for innovation failure (Zhong, 2018). According to signaling theory, when investors receive preparedness signals from entrepreneurs, they increase their failure tolerance by increasing the transparency of information between the two parties. This increased tolerance for failure can lead venture capitalists to more readily interpret the perception of value conveyed by preparedness signals and, in turn, to prefer to invest in early stage startups. In summary, venture capitalists’ willingness to invest increases after entrepreneurs signal to venture capitalists that they are ready to be invested in. Hence, we propose the following hypothesis:

*Hypothesis 1a (H1a)*: Entrepreneurial enthusiasm is positively related to venture capitalists’ willingness to invest.

*Hypothesis 1b (H1b)*: Entrepreneurial preparedness is positively related to venture capitalists’ willingness to invest.

### The Mediating Role of Relational Capital

The investment decision of venture capitalists is rational behavior (Yang et al., 2021); hence, scholars tend to choose mediating variables, such as perceived risk or perceived return, on the basis of transaction cost theory or agency theory to explain the mechanisms underlying entrepreneurial characteristics or behaviors that influence venture capitalists’ investment decision behavior or willingness (Arthurs and Busenitz, 2003). However, even though venture capitalists try to examine venture investments rationally, venture capitalists are still influenced by their cognitive biases (Linder and Sperber, 2020; Sanchez-Ruiz et al., 2021), especially when entrepreneurs release the interpersonal signal of entrepreneurial passion (Ciuchta et al., 2018). Transaction cost theory or agency theory cannot explain this phenomenon well, that is, there are certain shortcomings in explaining the formation mechanism of venture capitalists’ investment decisions around a single perspective. Thus, the current article introduces relational capital as a mediating variable from a relational perspective on the basis of signaling theory to reveal further the intrinsic mechanism by which entrepreneurial passion affects venture capitalists’ willingness to invest. As an essential concept derived from social capital, relational capital refers to assets acquired through building or leveraging relationships, including trust and credibility, norms and penalties, obligations and expectations, and recognizable identities. It emphasizes the personified aspect of social networks, which is related to socially connected actors, manifested as concrete, ongoing interpersonal relationships, specific relationships established by actors in the process of interaction (Nahapiet and Ghoshal, 1998). Venture capital is exposed to a high degree of uncertainty and risk, and venture capitalists’ investment decisions in entrepreneurs need intrinsic motivation to ensure their successful completion, and venture capitalists’ perceived relational capital may be one such driver. Venture capitalists with high perceived relational capital value relational resources that potentially funded entrepreneurs bring to the table, potentially linking themselves to the entrepreneurs, and enabling direct and indirect connections between the two parties in their social networks. In addition, the term sheet that provides the letter of intent to invest provides a degree of control mechanism to resolve contentious issues, such as valuation, compensation, and control rights. Once an entrepreneur accepts these control provisions, a degree of legal and emotional trust is created, thus increasing the willingness of early stage investors to invest in the entrepreneur. For instance, a high perceived control of venture capitalists directly affects the potential to invest in entrepreneurs (Drover et al., 2014). Thus, relational capital is closely related to venture capitalists’ investment decisions.

According to social capital theory, relational capital not only serves as a long-term asset with a return on investment to predict future benefit flows (Adler and Kwon, 2002) but also can be “constructed” and evaluated through the efforts of both parties (Kale et al., 2000). For example, scholars have suggested that relational capital can be measured through the constructs of trustworthiness and trust. According to signaling theory, the enthusiasm and preparedness signals conveyed by entrepreneurial passion tend to increase venture capitalists’ perception of the added value of entrepreneurs and startups, which, in turn, increases their credibility and trust in investing in entrepreneurs. Credibility is one of the most important criteria for venture capitalists to measure the value of an entrepreneur’s investment. However, in the early stages of entrepreneurship, due to incomplete and asymmetric information, venture capitalists need to rely on limited information to make decisions in limited time, which makes searching for specific signals and clues necessary for venture capitalists during their interactions with entrepreneurs (Daly and Davy, 2016). Combined with signaling theory, by introducing relational capital into the venture capital decision domain, entrepreneurial passion signals released by entrepreneurs can influence venture capitalists’ perception of relational capital at least at three levels: confidence building, trust-building, and emotional persuasion. First, the signals of enthusiasm sent by entrepreneurs can lead venture capitalists to have more confidence in entrepreneurs when the product and environment are uncertain (Zacharakis and Shepherd, 2001). The enthusiasm shows how motivated entrepreneurs are to overcoming rather than avoiding difficulties and obstacles (Cardon and Kirk, 2015). Second, preparedness signals from entrepreneurs can give venture capitalists the information they are looking for (Kirsch et al., 2009), including whether the entrepreneur has built an adequate plan and communicated it (Hill and Levenhagen, 1995) and if the entrepreneur is truly ready to receive investment. Trust, another important measure of relational capital, facilitates communication and information sharing between parties, thus encouraging the provision of resources and emotional support to promote relational capital (Liao and Welsch, 2005). Lastly, the trust that forms in the early stages of a business is mainly based on emotions, which is typical of pre-established relationships (Smith and Lohrke, 2008). The expression of emotions plays an important role, and positive emotions are persuasive (Hatfield et al., 1993; Baron, 2008). The positive emotion of entrepreneurial enthusiasm preemptively provides a venture capitalist with emotional trust in the interaction between the venture capitalist and the entrepreneur. Therefore, entrepreneurial passion can positively influence venture capitalists’ willingness to invest by enhancing venture capitalists’ relational capital as well. Hence, we propose the following hypothesis:

*Hypothesis 2a (H2a)*: Entrepreneurial enthusiasm promotes relational capital, which, in turn, increases venture capitalists’ willingness to invest.

*Hypothesis 2b (H2b)*: Entrepreneurial preparedness promotes relational capital, which, in turn, increases venture capitalists’ willingness to invest.

### The Moderating Role of Relational Capital

In our study, in addition to acting as a mediating variable, relational capital may reinforce the effect of entrepreneurial passion on venture capitalists’ willingness to invest. Relational capital motivates the willingness of both parties to cooperate. Scholars find that relational capital is the “bridge” between supplier development and relationship benefits. Without relational capital, there is not provide the benefits of capability development but instead has detrimental effects on the supplier management system (Blonska et al., 2013). Entrepreneurial passion reflects the conscious and intense emotional experiences that entrepreneurs develop during the entrepreneurial process. This experience can be transmitted to entrepreneurial stakeholders through emotional contagion (Cardon, 2008; Cardon et al., 2009), causing stakeholders to endorse the entrepreneurial idea of the entrepreneur, which, in turn, gives legitimacy to the entrepreneurial venture. According to signaling theory, we believe that a gap exists between the entrepreneurial passion (entrepreneurial enthusiasm and entrepreneurial preparedness) released by entrepreneurs and venture capitalists’ willingness to invest. Thus, the ability of passion signals released by entrepreneurs to promote venture investors’ willingness to invest also depends on venture capitalists’ perception of relational capital. Previous research agrees that perceived entrepreneurial passion is considered when venture capitalists make investment decisions (Elsbach and Kramer, 2003). Relational capital implies that venture capitalists consider the potential return on investment benefits that entrepreneurs and their ventures bring to themselves when making decision evaluations of entrepreneurs. A venture capitalist’s cognitive interpretation of an entrepreneur’s signal of releasing entrepreneurial passion may lead to a willingness to return the entrepreneur’s investment. Therefore, we further explore the role of relational capital as a moderating variable, arguing that it strengthens the relationship between entrepreneurial passion and venture capitalists’ willingness to invest. Venture capitalists with high relational capital perception may be more sensitive to the entrepreneurial passion released by entrepreneurs, and they are better at capturing the potential value information conveyed by such entrepreneurial passion signals. For example, emotional expressions (such as verbal expressions, facial expressions, and body language) and cognitive behaviors (such as business plan and presentation preparation) that an entrepreneur displays during the business presentation represent the entrepreneur’s confidence and commitment to the business and reflect the state of the business to a certain extent (Chen et al., 2009). This state of the business may be what venture capitalists look for when making investment decisions. Thus, entrepreneurial passion signals may impress venture capitalists even more. On the basis of “reciprocity principle,” relational capital may reduce the perceived risk of venture capitalists and encourage entrepreneurial returns on venture capital investments, which can significantly facilitate venture capitalists’ investment intentions.

By contrast, venture capitalists with low relational capital may be more rational about the entrepreneurial passion unleashed by entrepreneurs. They may not be easily contagious by entrepreneurial passion and are even less likely to accept entrepreneurs. Scholars identified compulsive enthusiasm, which is false enthusiasm that entrepreneurs will show to be favored by investors (Ho and Pollack, 2014). Entrepreneur preparedness signals convey mainly an entrepreneur’s growth and ideas for the future of the business. However, because of the incomplete alignment of target interests, investors also worry about the real situation of the entrepreneur and his team in the future cooperation. The investment of both parties is relationship-specific; hence, establishing effective mechanisms to discourage “free-rider” behavior is necessary, and relational capital provides this reciprocity. However, the perception of reciprocity among venture capitalists with low relational capital may have differences. When interpreting signals of entrepreneurial passion, venture capitalists may make fewer investment decisions due to a lack of willingness to invest on the basis of reward. Hence, we propose the following hypothesis:

*Hypothesis 3a (H3a)*: Relational capital reinforces the relationship between entrepreneurial enthusiasm and the willingness of venture capitalists to invest.

*Hypothesis 3b (H3b)*: Relational capital reinforces the relationship between entrepreneurial preparedness and the willingness of venture capitalists to invest.

**Figure 1:**
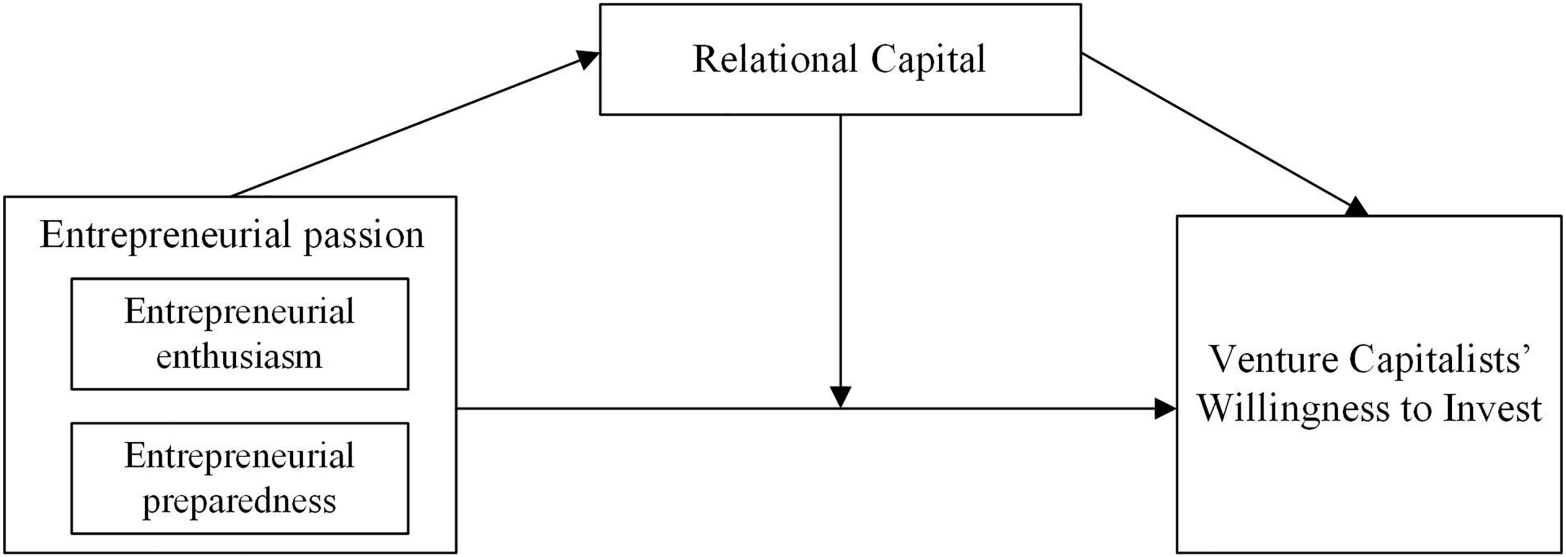
Theoretical model.

Figure 1 shows the theoretical model of this research based on the above theoretical view and research hypotheses.

## Materials and Methods

### Sample and Procedure

The empirical data selected for this study come from China’s first venture capital reality show “CHINESE DRAGON’S DEN,” a program in which five venture capitalists worth over $100 billion negotiate financing with entrepreneurs. In the show, entrepreneurs pitch their companies to five potential investors, hoping to compel any one of the venture capitalists to meet their expectations for investment capital. The flow of the show is as follows: first, the entrepreneurs introduce their names, project names, and expected amount of funding, and then present their projects. Afterward, five venture capitalists ask the entrepreneurs questions. This interaction continues until each of the five venture capitalists makes their respective investment decisions. Ultimately, the interactions between entrepreneurs and venture capitalists were used to produce segments for the show (25 episodes of 45min each). We chose this show as the data source for two reasons. First, the information is publicly available and easily accessible. Second, the information relevant to investors’ decisions is authentic and reliable. The producers of the show emphasize that the negotiations between venture capitalists and entrepreneurs for business financing are real. In the show, the startup investors’ decisions reflect their true investment intentions. To test our hypothesis, we used the observational interaction, which was optimized by Maxwell and Lévesque (2014) in accordance with the verbal protocol analysis pioneered by Bakeman and Gottman (1997). This approach is based on observing the interaction between the parties and allows trained raters to extract useful information for further analysis (Maxwell and Lévesque, 2014). First, select five trained raters from the research team to code the video of the entrepreneurs’ live presentations. The raters were primarily responsible for observing the interaction process between each entrepreneur and venture capitalists. This observation continued until all five venture capitalists exited, or one or more venture capitalists made their investment offers. We measured all items on a 5-point Likert scale except for an objective measure of venture capitalists’ willingness to invest. Second, five raters evaluated 79 videos over 4days. In the full scale, the inter-rater reliability among the five raters was 0.802, indicating inter-rater agreement. Finally, we aggregated the codes of the five raters and performed model validation on this basis (see Table 1).

**Table 1:** Deals total factor scores, selection stage disposition, and industry types.

Number	Industry type	Score	Select	Number	Industry type	Score	Select
1	Life Services	44.6	NO	41	E-Commerce	53.8	YES
2	Enterprise Service	52.6	YES	42	E-Commerce	44	NO
3	Life Services	53.4	YES	43	Artificial Intelligence	57.8	YES
4	E-Commerce	59.6	YES	44	Life Services	44.8	NO
5	Life Services	49.2	NO	45	Traditional Industry	50.4	NO
6	Sports Industry	59.6	YES	46	Sports Industry	44.2	NO
7	Culture and Recreation	45.6	NO	47	Culture and Recreation	56	YES
8	Healthcare	51.2	YES	48	Tourism Industry	43.8	NO
9	E-Commerce	53.4	YES	49	Culture and Recreation	41.6	NO
10	Traditional Industry	55.4	YES	50	E-Commerce	55.6	YES
11	E-Commerce	39.4	NO	51	Culture and Recreation	36.4	NO
12	Education and Training	55.8	YES	52	Life Services	55	YES
13	E-Commerce	58.4	YES	53	Culture and Recreation	42.4	NO
14	Life Services	65.6	YES	54	E-Commerce	45.8	NO
15	Traditional Industry	40.4	NO	55	Culture and Recreation	60	YES
16	E-Commerce	59.8	YES	56	Healthcare	47.8	YES
17	E-Commerce	53.4	YES	57	Artificial Intelligence	44.8	NO
18	Traditional Industry	59.6	YES	58	Education and Training	66.8	YES
19	Tourism Industry	43.6	NO	59	E-Commerce	46.2	NO
20	Life Services	49.2	YES	60	Traditional Industry	49	NO
21	Education and Training	43	NO	61	Education and Training	41.8	NO
22	Education and Training	46	YES	62	Education and Training	50.4	NO
23	Traditional Industry	55	YES	63	Culture and Recreation	56.6	YES
24	Life Services	48.2	NO	64	E-Commerce	48.8	NO
25	Healthcare	42.2	NO	65	Artificial Intelligence	45.4	NO
26	Life Services	53.6	YES	66	E-Commerce	63	YES
27	Intelligent Manufacturing	43.8	NO	67	Intelligent Manufacturing	36.6	NO
28	E-Commerce	61.2	YES	68	E-Commerce	42.6	NO
29	Intelligent Manufacturing	51.8	YES	69	Life Services	39.4	NO
30	Education and Training	52.4	NO	70	Artificial Intelligence	45	NO
31	Big Data	68.6	YES	71	Traditional Industry	52.2	YES
32	Education and Training	52	YES	72	E-Commerce	41.6	NO
33	Culture and Recreation	46.4	NO	73	Life Services	49	NO
34	E-Commerce	40.4	NO	74	Intelligent Manufacturing	47.8	NO
35	Education and Training	34.6	NO	75	Life Services	52.8	YES
36	Traditional Industry	46.4	NO	76	Life Services	37.4	NO
37	Education and Training	48.2	NO	77	E-Commerce	47.4	NO
38	Life Services	48.6	NO	78	Artificial Intelligence	48.8	NO
39	E-Commerce	42.2	NO	79	Culture and Recreation	51.6	YES
40	Culture and Recreation	43.8	NO				

### Measures

#### Entrepreneurial Passion

Entrepreneurial passion was assessed according to an adaptation of the enthusiasm and preparedness scale developed by Chen et al. (2009). Items to assess enthusiasm include whether the entrepreneur has energetic body movements and vivid facial expressions. Preparedness items include whether the entrepreneur’s presentation is substantive, thoughtful and in-depth, coherent, and logical (see Table 2). The assessments were all set within a 5-point range. The Cronbach’s α was 0.844.

**Table 2:** Measuring scale of variables.

Factor	Key questions
Entrepreneurial enthusiasm (Chen et al., 2009)	The presenter(s) had energetic body movements.
	The presenter(s) showed animated facial expression.
	The presenter’s face lit up when he/she or he talked.
	The presenter(s) talked with varied tone and pitch.
Entrepreneurial preparedness (Chen et al., 2009)	The presentation content had substance.
	The presentation was thoughtful and in-depth.
	The presentation was coherent and logical.
	The presenter(s) articulated the relationship between his b-plan and the broader context.
	The presenter(s) cited facts to support his/her arguments.
Relational capital (Blonska et al., 2013)	I believe that entrepreneurs will consider the best interests of investors.
	Entrepreneurs can be counted on to keep their promises.
	The investor–entrepreneur relationship can be defined as “mutually beneficial.”
	Venture capitalists look forward to working with entrepreneurs.
Willingness to invest (Ciuchta et al., 2018)	Would you personally invest in this entrepreneur’s new venture?
	Would you personally recommend to other persons that they make an investment in this venture?

#### Relational Capital

Relational capital includes the three dimensions of trust, reciprocity, and affective commitment, all of which reflect the uniqueness of the relationship, that is, how the venture capitalist views the relationship. Affective commitment is the tendency of a supplier to maintain the relationship (Kumar et al., 1994). Reciprocity represents the sense of debt and obligation experienced by both parties toward the relationship in the future (Palmatier, 2008; Hoppner and Griffith, 2011). Trust is the degree to which partners expect from each other, an expectation that is not a selfish act, but a promise kept (Kaufman et al., 2006). Therefore, the study based on the relational capital scale compiled by Blonska et al. (2013), identifies five items to assess the entrepreneur’s relational capital, including whether the entrepreneur can be expected to keep his or her word, and whether the cooperation with the entrepreneur is expected (see Table 2). The assessments were all set within a 5-point range. The Cronbach’s α was 0.866.

#### Willingness to Invest

As a dependent variable, the value of venture capitalists’ willingness to invest is supposed to be a continuous variable. However, this variable could not be directly observed in the actual operation of data collection in this paper. Therefore, we use the investment decision behavior of the five venture capitalists in the show instead. Referring to Ciuchta et al. (2018), we set the range of 2 for the measure of willingness to invest. By observing “whether the venture capitalist would invest in the entrepreneur’s project personally” and “whether the venture capitalist would recommend others to invest in the project,” we consider 1 to be reluctant and 2 to be willing (see Table 2). The Cronbach’s α was 0.861.

#### Control Variables

Individual differences in entrepreneurs have an impact on the investment decisions of venture capitalists (Drover et al., 2014; Parhankangas and Renko, 2017). Therefore, on the basis of previous studies, the subsequent empirical analysis of this study needs to control for variables, such as age, gender, and the presence or absence of entrepreneurial experience of entrepreneurs. Male entrepreneurs are coded as 0 and female entrepreneurs are coded as 1. Age was coded according to the following groups: 1=25 years old and blew, 2=26–35, 3=36–45, 4=46–55, 5=56 years old and above. In addition, we control for whether the entrepreneur has previous entrepreneurial experience, where having entrepreneurial experience is 1 and vice versa is 0.

## Results

### Confirmatory Factor Analyses

The study used validated factor analysis to examine the discriminant validity of entrepreneurial passion (including enthusiasm and preparedness), relational capital, and venture capitalists’ willingness to invest. Table 3 shows the results, which indicate that the fit of the four-factor model aptitude indicators is significantly better than that of other factor models, such as the three-factor and two-factor models. The indicators of the model were as: χ^2^/df=1.337, CFI=0.972, TLI=0.964, and RMSEA=0.066. These indicators indicate that the four main variables involved have better discriminant validity in this study.

**Table 3:** Results of confirmatory factor analysis of the measurement models.

Model	*χ* ^2^	*df*	*χ* ^2^ */df*	*CFI*	*TLI*	*RMSEA*
Four-factor model	109.645	82	1.337	0.972	0.964	0.066
Three-factor model	145.242	85	1.709	0.939	0.925	0.095
Two-factor model	153.620	87	1.766	0.933	0.919	0.099
One-factor model	156.530	88	1.779	0.931	0.917	0.100

*N*=79. En, entrepreneurial enthusiasm; Pre, entrepreneurial preparedness; Re, Relational capital; and Vc, venture capitalists’ willingness to invest. In the three-factor model, items of En and Pre were loaded on one factor. In the two-factor model, items of En, Pre, and Vc were loaded on one factor.

### Preliminary Analyses

Table 4 shows the means, standard deviations, and correlation coefficients of the study variables. Enthusiasm (*r*=0.322, *p*<0.01) and preparedness (*r*=0.866, *p*<0.01) showed a significant positive relationship with venture capitalists’ willingness to invest. Therefore, hypotheses 1 and 2 were initially supported. Relational capital was significantly and positively associated with enthusiasm (*r*=0.315, *p*<0.01), preparedness (*r*=0.868, *p*<0.01), and willingness to invest (*r*=0.881, *p*<0.01). Therefore, hypothesis 3a and hypothesis 3b were initially supported.

**Table 4:** Means, standard deviations, and correlations for all variables.

Variables	Mean	SD	1	2	3	4	5	6
1. En	3.14	0.467						
2. Pre	2.83	0.740	0.244^*^					
3. Re	2.54	0.499	0.315^**^	0.868^**^				
4. Vc	1.31	0.327	0.322^**^	0.866^**^	0.881^**^			
5. Gender	0.35	0.481	0.323^**^	0.085	0.147	0.116		
6. Age	2.10	0.761	−0.155	0.060	0.051	−0.008	−0.204	
7. Experience	1.24	0.430	−0.130	0.000	−0.003	−0.029	−0.107	0.395^**^

Significance codes:

* *p*<0.05;

** *p*<0.01; ***p<0.001.

### Analyses of the Main Effect and Mediating Effect

We test sequentially whether relational capital mediates the relationship between entrepreneurial enthusiasm, preparedness, and venture capitalists’ willingness to invest. Table 5 shows the results of the analysis. First, model M1 was constructed by including relational capital as the dependent variable and the control variables (gender, age, and entrepreneurial experience) in the equation. According to model M1, the independent variables entrepreneurial enthusiasm and entrepreneurial preparedness are added in turn to construct model M2 and model M3, respectively, so as to test the relationship between entrepreneurial enthusiasm, entrepreneurial preparedness, and relational capital. As shown by model M2 and model M3, entrepreneurial enthusiasm has a significant positive effect on relational capital (M2, *β*=0.309, *p*<0.01); entrepreneurial preparedness has a significant positive effect on relational capital (M3, *β*=0.861, *p*<0.001).

**Table 5:** Mediation effect test of relational capital.

Variables	Re			Vc					
	M1	M2	M3	M4	M5	M6	M7	M8	M9
Gender	0.164	0.071	0.078	0.119	0.021	0.032	−0.027	−0.040	−0.009
Age	0.093	0.114	0.016	0.027	0.048	−0.051	−0.056	−0.051	−0.059
Experience	−0.023	0.000	−0.001	−0.027	−0.004	−0.006	−0.007	−0.004	−0.005
En		0.309^**^			0.322^**^			0.052	
Pre			0.861^***^			0.866^***^			0.410^***^
Re							0.888^***^	0.873^***^	0.529^***^
R^2^	0.029	0.114	0.760	0.014	0.106	0.754	0.780	0.782	0.821
∆R^2^	—	0.085	0.731	—	0.092	0.739	0.766	0.768	0.807
F	0.748	2.370	58.474^***^	0.364	2.191	56.621^***^	65.567^***^	52.404^***^	66.985^***^

Significance codes: *p<0.05;

** *p*<0.01;

*** *p*<0.001.

Second, model M4 is constructed by including venture capitalists’ willingness to invest as the dependent variable and including the control variables into the equation. Based on model M4, the independent variables entrepreneurial enthusiasm and entrepreneurial preparedness are added in turn to construct models M5 and M6, respectively, to test the relationship between entrepreneurial enthusiasm, entrepreneurial preparedness, and venture capitalists’ willingness to invest. Model M5 indicates that entrepreneurial enthusiasm has a significant positive effect on venture capitalists’ willingness to invest (M5, *β*=0.322, *p*<0.01). Therefore, H1a is further supported. Model M6 shows that entrepreneurial preparedness has a significant positive effect on venture capitalists’ willingness to invest (M6, *β*=0.866, *p*<0.001). H1b is further supported. Again, on the basis of model M4, model M7 is constructed with venture capitalists’ willingness to invest as the dependent variable and by adding the mediating variable relational capital to test the relationship between relational capital and venture capitalists’ willingness to invest. As shown by model M7, relational capital has a significant positive effect on venture capitalists’ willingness to invest (M7, *β*=0.888, *p*<0.001).

Third, the willingness of venture capitalists to invest as the dependent variable, control variables, independent variables (entrepreneurial enthusiasm and entrepreneurial preparedness), and mediating variables (relational capital) are simultaneously entered into the regression equation to construct model M8 and model M9. As shown by model M8, relational capital still has a positive effect on venture capitalists’ willingness to invest (M8, *β*=0.873, *p*<0.001), but the effect of entrepreneurial enthusiasm on venture capitalists’ willingness to invest is significantly lower (M8, *β*=0.052, *p*>0.05). This result indicates that relational capital plays a fully mediating role between entrepreneurial enthusiasm and venture capitalists’ willingness to invest, that is, H2a is supported. As shown by model M9, relational capital still has a positive effect on venture capitalists’ willingness to invest (M9, *β*=0.529, *p*<0.001), but the effect of preparedness on venture capitalists’ willingness to invest decreases (M9, *β*=0.410, *p*<0.001). Therefore, relational capital partially mediates between entrepreneurial preparedness and venture capitalists’ willingness to invest, that is, H2b is supported.

### Analyses of the Moderating Effect

Finally, with venture capitalists’ willingness to invest as the dependent variable, control variables, independent variables (entrepreneurial enthusiasm and entrepreneurial preparedness), moderating variables, and interaction terms were entered into the regression equation. Models M10, M11, M12, and M13 were constructed to test sequentially whether relational capital moderates the relationship between entrepreneurial enthusiasm and venture capitalists’ willingness to invest. Table 6 shows the results. Model M12 and model M13 show that the interaction term between entrepreneurial preparedness and relational capital (M13, *β*=0.161, *p*<0.01) has an effect on venture capitalists’ willingness to invest, indicating that relational capital moderates the relationship between entrepreneurial preparedness and venture capitalists’ willingness to invest. H3b is supported. The interaction term between entrepreneurial enthusiasm and relational capital (M12, *β*=0.011, *p*>0.05) was not validated for H3a.

**Table 6:** Mediation effect test of relational capital.

Variables	Vc			
	M10	M11	M12	M13
Gender	−0.040	−0.009	−0.041	−0.015
Age	−0.051	−0.059	−0.050	−0.038
Experience	−0.004	−0.005	−0.003	−0.039
Pa	0.052		0.052	
Pre		0.410^***^		0.447^***^
Re	0.873^***^	0.529^***^	0.870^***^	0.455^***^
Pa×Re			0.011	
Pre×Re				0.161^**^
R^2^	0.782	0.821	0.782	0.844
∆ R^2^	0.768	0.807	0.768	0.830
F	52.404^***^	66.985^***^	43.097^***^	64.738^***^

Significance codes: *p<0.05;

** *p*<0.01;

*** *p*<0.001.

**Figure 2:**
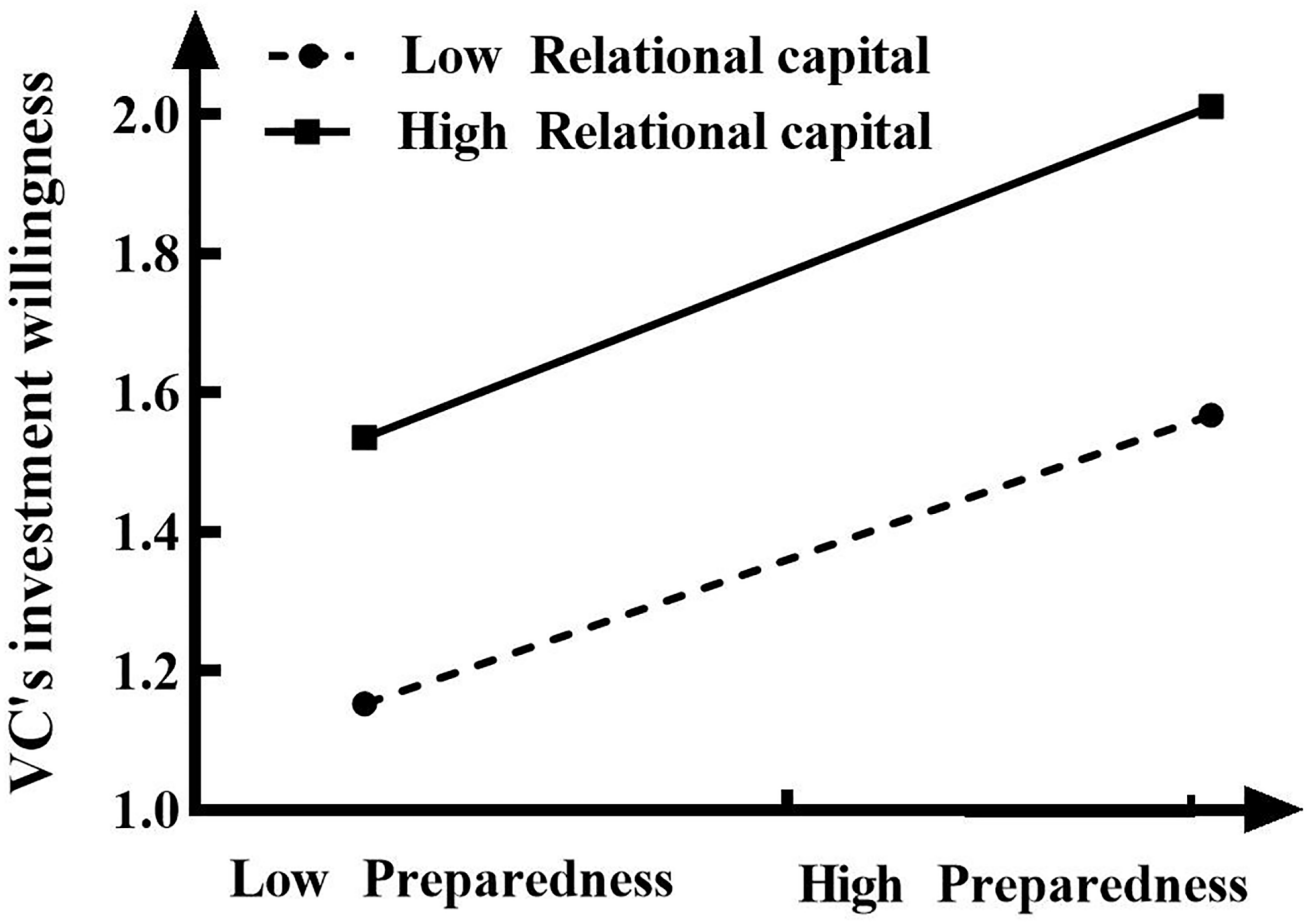
Interactive effect of the relational capital and entrepreneurial preparedness on venture capitalists’ willingness to invest.

To further test whether the moderating effect of relational capital on the relationship between entrepreneurial preparedness and venture capitalists’ willingness to invest is consistent with the expectations of the previous hypothesis, the values of the mean plus or minus one standard deviation of entrepreneurial preparedness and relational capital were added in the regression model, and the interaction effects were plotted (see Figure 2). Figure 2 shows that the slope of the regression is significantly positive at low levels of relational capital (*γ*=0.425, *t*=4.377, *p*<0.001), whereas it remains significant at high levels of relational capital (*γ*=0.571, *t*=5.385, *p*<0.001). This result suggests that the positive association between entrepreneurial preparedness and venture capitalists’ willingness to invest is strong for entrepreneurs with high relational capital, whereas the positive association between entrepreneurial preparedness and venture capitalists’ willingness to invest is weak for entrepreneurs with low relational capital. Therefore, H3b is supported.

## Discussion

### Theoretical Contributions

The study expands and adds depth to the literature and research related to entrepreneurial passion, signaling theory, and relational capital in the following ways.

First, the study explores the potential impact of entrepreneurial passion on venture capitalists’ willingness to invest from a signaling theory perspective, enriching the theoretical study of entrepreneurial passion in the field of venture capital. The study introduces signaling theory to explore the relationship between investors and entrepreneurs. Venture capitalists, to some extent, consider different types of signals that entrepreneurs may convey to make investment decisions when assessing the financing potential of a startup (Ko and McKelvie, 2018; Calic and Shevchenko, 2020). Potential stakeholders may vary in the extent to which they perceive motivational cues, or which specific cues they focus on that may ultimately influence their decision to participate in a startup (Cardon et al., 2017). Much research has been conducted by scholars in the field of entrepreneurship around the factors influencing investors’ investment decisions. However, except for a few studies (Chen et al., 2009; Cardon and Kirk, 2015), not much literature has discussed how entrepreneurial passion affects venture capitalists’ investment decisions, and even less research has examined the process effects of entrepreneurial passion on venture capitalists’ investment decisions. Unlike most previous studies that have examined entrepreneurial passion as a separate construct (Mitteness et al., 2012; Eddleston et al., 2016; Hubner et al., 2020), this article distinguishes passion for the enthusiasm construct according to Cardon’s and Chen’s understanding: entrepreneurial enthusiasm and how entrepreneurial preparedness affects venture capitalists’ willingness to invest. Specifically, in the early stage of entrepreneurship, when entrepreneurs are interacting with venture capitalists, the more enthusiasm and preparedness entrepreneurs convey to venture capitalists, the more willing the venture capitalists are to invest in the entrepreneurs. However, Chen et al. (2009) found that enthusiasm did not have a significant impact on investors’ investment decisions, suggesting that investors’ previous experiences and a large amount of monetary investment may lead to cautious decision making. We can only speculate on other possible explanations. First, the data for this study came from China. The different cultural contexts in China and the United States ultimately lead to different results. China is a “guanxi” culture society and is more likely to make investment decisions because of the excellence of entrepreneurs themselves. Second, the differences may also be due to the scope of the data. The data came from the scope of the program presented. In the broader realm of venture capital investment, different constraints may lead to different findings. All possibilities await more research to sort out the role of passion for investment decisions. In a word, this study focuses on two dimensions of entrepreneurial passion as an interpersonal signal to reveal the impact of entrepreneurial enthusiasm and entrepreneurial preparedness on venture capitalists’ willingness to invest, which is a good extension and complement to the existing research on entrepreneurial passion in the field of venture capital.

Second, the study finds that entrepreneurial passion can have a facilitating effect on venture capitalists’ willingness to invest through the mediation of relational capital. Most previous studies have acknowledged the positive impact of entrepreneurial passion on investors’ investment decisions, but only a few studies have explored the underlying mechanisms (Chen et al., 2009; Cardon et al., 2017). The results of the study showed that relational capital fully mediates the relationship between entrepreneurial enthusiasm and venture capitalists’ willingness to invest. We believe the effectiveness of the signal also depends on the perception and interpretation of the venture capitalist. In general, prior investment experience will make a venture capitalist more aware of the potential outcomes associated with an entrepreneur’s signals. With this heightened awareness, the venture capitalist will be particularly sensitive to signals. The passion shown by entrepreneurs is also likely to give rise to suspicion of “performance” on the part of venture capitalists, and the presence of relational capital largely reduces such suspicion. The entrepreneurial passion signals transmitted by the entrepreneur to the venture capitalist can convey additional information to the venture capitalist, and this information can facilitate the construction of perceived relational capital by the venture capitalist. This perceived relational capital emotionally increases the venture capitalist’s trust in the entrepreneur, which, in turn, increases the venture capitalist’s willingness to invest. According to signaling theory, relational capital fully mediates the relationship between entrepreneurial enthusiasm and venture capitalists’ willingness to invest. We argue that entrepreneurial enthusiasm is, to some extent, more than a fully positive emotion. Unlike entrepreneurial preparedness, the enthusiasm shown by entrepreneurs is also likely to give rise to suspicion of “performance” on the part of venture capitalists. The presence of relational capital largely reduces this suspicion and thus positively influences the willingness of venture capitalists to invest.

Furthermore, this study extends the broader understanding and application of relational capital by introducing the concept of relational capital to this stage before investors and entrepreneurs reach a partnership. Most previous studies on relational capital have focused on supplier-buyer partnerships (Blonska et al., 2013), but only a few studies have focused on how entrepreneurs build and leverage relational capital in the early stages. This study considers relational capital as a bridge to build a relationship between entrepreneurs and venture capitalists. Starting from the entrepreneurial passion of entrepreneurs, we explore in-depth the intrinsic mechanism of the impact of entrepreneurial passion on venture capitalists’ willingness to invest and reveal the important role played by relational capital.

Third, the study reveals the borderline role of relational capital between entrepreneurial passion and venture capitalists’ willingness to invest. On the basis of signaling theory, this study finds that relational capital positively facilitates the relationship between entrepreneurial preparedness and venture capitalists’ willingness to invest. The entrepreneurial preparedness signals of startups with high relational capital are better perceived by venture capitalists and enhance venture capitalists’ willingness to invest. However, inconsistent with the expected results of this paper, startup entrepreneurial enthusiasm with high relational capital did not facilitate venture capitalists’ willingness to invest. As discussed previously, this study confirms the facilitative effect of enthusiasm on venture capitalists’ willingness to invest, whereas relational capital does not facilitate this relationship. Considering the possible reason that venture capitalists are experienced in investing, it is possible that entrepreneurs with high relational capital and high levels of passion are more likely to be aware of the dangers of using emotional expressions; thus, they are less likely to be able to control them. Third, the ineffective moderating role of relational capital between enthusiasm and venture capitalists’ willingness to invest provides direction for further research. The moderating role of relational capital demonstrates its importance in the field of venture capital and provides a further deepening of the explanatory framework for influencing investors’ investment decisions.

### Practical Implications

The findings of this study have managerial implications. First, this study highlights the important role of entrepreneurial passion as an interpersonal signal during the interaction between entrepreneurs and venture capitalists. In our study, entrepreneurial enthusiasm and business preparedness are important factors for venture capitalists in determining their willingness to invest. The results of the study show that the higher the enthusiasm and preparedness of entrepreneurs, the stronger the willingness of venture capitalists to invest. On the one hand, entrepreneurs need to give their business a meaningful background and a clear positioning. On the other hand, entrepreneurs have a clear and explicit plan for the future development of their business, which is an important aspect to convince venture capitalists to invest and is a reflection of the enthusiasm and preparedness that entrepreneurs put into their business.

Second, startups need to recognize the positive role of fully utilizing relational capital in obtaining investment from venture capitalists. The results of the study indicate that the relational capital of entrepreneurs perceived by venture capitalists occupies an important position in the relationship between entrepreneurial enthusiasm and venture capitalists’ willingness to invest. It plays a completely mediating role between entrepreneurial enthusiasm and venture capitalists’ willingness to invest. This finding reveals to entrepreneurs that when startups are in the risky early stages, focusing not only on product development and promotion but also on cultivating the relational capital of the business is important, because relational capital is directly related to the trust generated by venture capitalists. Entrepreneurs must be aware of the importance of how to build appropriate relational capital with different stakeholder groups not only in terms of the number of relationships, but also the quality of relationships. Entrepreneurs should give conscious effort to build relationships and be able to cultivate them appropriately.

### Research Limitations and Future Research Directions

Although some valuable conclusions were obtained in this paper, it has shortcomings. First, this study collects data based on observational interaction, which is coded by watching a venture capital type show. Although the method has some reliability, due to the limitation of the number of videos provided by this show, the lack of statistics related to venture capitalists and the limitations of the characteristics of the dependent variable makes it difficult to rely on the data to provide robustness tests for the conclusions in this paper. Second, the study shows that entrepreneurial enthusiasm has a significant positive effect on venture capitalists’ willingness to invest. This result is inconsistent with Chen et al. (2009) finding that the effect of entrepreneurial enthusiasm on investors’ investment decisions is not significant. Considering that the data used in this study are from China, whether the differences in cultural contexts of the United States and China ultimately lead to different results needs to be further investigated in subsequent studies. Third, unlike what is expected in the paper, relational capital does not have a significant moderating role in the relationship between entrepreneurial enthusiasm and venture capitalists’ willingness to invest. Venture capitalists do not increase their trust in entrepreneurial enthusiasm signals due to the high relational capital of startups, which, in turn, does not contribute to venture capitalists’ investment decisions. The deeper reasons for this finding are yet to be explored.

## Conclusion

On the basis of signaling theory, this study investigates the relationship between entrepreneurial passion and venture capitalists’ willingness to invest using 79 interactions sample between entrepreneurs and venture capitalists. The focus is on the mediating and moderating role of relational capital. The findings show that entrepreneurial enthusiasm and preparedness have a significant positive effect on venture capitalists’ willingness to invest. Relational capital mediates the relationship between entrepreneurial enthusiasm and preparedness and venture capitalists’ willingness to invest. Furthermore, relational capital positively moderates the relationship between entrepreneurial preparedness and venture capitalists’ willingness to invest. The study provides a new approach to explore the mechanism of influence and the boundary of action between entrepreneurial passion and venture capitalists’ willingness to invest.

## Data Availability Statement

The raw data supporting the conclusions of this article will be made available by the authors, without undue reservation.

## Ethics Statement

Ethical review and approval were not required for the study on human participants in accordance with the local legislation and institutional requirements. The patients/participants provided their written informed consent to participate in this study.

## Author Contributions

HY created and designed the theoretical model and wrote the first manuscript. HS conceptualized the study, collected the data, and wrote the first manuscript. YW and LZ analyzed the data and improved the manuscript. SX collected and analyzed the data. All authors contributed to the article and approved the submitted version.

## Funding

This research was funded by National Social Science Foundation of China (No. 19BSH110).

## Conflict of Interest

The authors declare that the research was conducted in the absence of any commercial or financial relationships that could be construed as a potential conflict of interest.

## Publisher’s Note

All claims expressed in this article are solely those of the authors and do not necessarily represent those of their affiliated organizations, or those of the publisher, the editors and the reviewers. Any product that may be evaluated in this article, or claim that may be made by its manufacturer, is not guaranteed or endorsed by the publisher.

## References

[ref1] Abdullah M. I. Dechun H. Ali M. Usman M. (2019). Ethical leadership and knowledge hiding: a moderated mediation model of relational social capital, and instrumental thinking. Front. Psychol. 10:2403. doi: 10.3389/fpsyg.2019.02403, PMID: 31708841 PMC6823209

[ref2] Adler P. S. Kwon S. W. (2002). Social capital: prospects for a new concept. Acad. Manag. Rev. 27, 17–40. doi: 10.2307/4134367

[ref3] Amit R. Glosten L. Muller E. (1990). Entrepreneurial ability, venture investments, and risk sharing. Manag. Sci. 36, 1233–1246. doi: 10.1287/mnsc.36.10.1233

[ref4] Arthurs J. D. Busenitz L. W. (2003). The boundaries and limitations of agency theory and stewardship theory in the venture capitalist/entrepreneur relationship. Entrep. Theory Pract. 28, 145–162. doi: 10.1046/j.1540-6520.2003.00036.x

[ref5] Bakeman R. Gottman J. M. (1997). Observing Interaction: An Introduction to Sequential Analysis. Cambridge, UK: Cambridge University Press.

[ref6] Baron R. A. (2008). The role of affect in the entrepreneurial process. Acad. Manag. Rev. 33, 328–340. doi: 10.5465/amr.2008.31193166

[ref7] Baum J. R. Locke E. A. (2004). The relationship of entrepreneurial traits,skill, and motivation to subsequent venture growth. J. Appl. Psychol. 89, 587–598. doi: 10.1037/0021-9010.89.4.587, PMID: 15327346

[ref8] Bi S. Liu Z. Usman K. (2017). The influence of online information on investing decisions of reward-based crowdfunding. J. Bus. Ventur. 71, 10–18. doi: 10.1016/j.jbusres.2016.10.001

[ref9] Blonska A. Storey C. Rozemeijer F. Wetzels M. de Ruyter K. (2013). Decomposing the effect of supplier development on relationship benefits: The role of relational capital. Ind. Mark. Manag. 42, 1295–1306. doi: 10.1016/j.indmarman.2013.06.007

[ref10] Bower G. H. (1981). Mood and memory. Am. Psychol. 36, 129–148. doi: 10.1037/0003-066X.36.2.129, PMID: 7224324

[ref11] Busenitz L. W. Fiet J. O. Moesel D. D. (2005). Signaling in venture capitalist—new venture team funding decisions: does it indicate long-term venture outcomes? Entrep. Theory Pract. 29, 1–12. doi: 10.1111/j.1540-6520.2005.00066.x

[ref12] Calic G. Shevchenko A. (2020). How signal intensity of behavioral orientations affects crowdfunding performance: The role of entrepreneurial orientation in crowdfunding business ventures. J. Bus. Ventur. 115, 204–220. doi: 10.1016/j.jbusres.2020.04.060

[ref13] Cardon M. S. (2008). Is passion contagious? The transference of entrepreneurialpassion to employees. Hum. Resour. Manag. Rev. 18, 77–86. doi: 10.1016/j.hrmr.2008.04.001

[ref14] Cardon M. S. Kirk C. P. (2015). Entrepreneurial passion as mediator of the self–efficacy to persistence relationship. Entrep. Theory Pract. 39, 1027–1050. doi: 10.1111/etap.12089

[ref15] Cardon M. S. Mitteness C. Sudek R. (2017). Motivational cues and angelinvesting: interactions among enthusiasm, preparedness, and commitment. Entrep. Theory Pract. 41, 1057–1085. doi: 10.1111/etap.12255

[ref16] Cardon M. S. Wincent J. Singh J. Drnovsek M. (2009). The nature andexperience of entrepreneurial passion. Acad. Manag. Rev. 34, 511–532. doi: 10.5465/amr.2009.40633190

[ref17] Chen X. Yao X. Kotha S. (2009). Entrepreneurial passion and preparedness in business plan presentations. Acad. Manag. J. 52, 199–214. doi: 10.5465/AMJ.2009

[ref18] Ciuchta M. P. Letwin C. Stevenson R. McMahon S. Huvaj M. N. (2018). Betting on the coachable entrepreneur: Signaling and social exchange in entrepreneurial pitches. Entrep. Theory Pract. 42, 860–885. doi: 10.1177/1042258717725520

[ref19] Claessens S. Tzioumis K. (2006). Ownership and financing structures of listed and large non-listed corporations. Corp. Gov. 14, 266–276. doi: 10.1111/j.1467-8683.2006.00506.x

[ref20] Collewaert V. Manigart S. (2016). Valuation of angel-backed companies: The role of investor human capital. J. Small Bus. Manag. 54, 356–372. doi: 10.1111/jsbm.12150

[ref21] Connelly B. L. Certo S. T. Ireland R. D. Reutzel C. R. (2011). Signaling theory: A review and assessment. J. Manag. 37, 39–67. doi: 10.1177/0149206310388419

[ref004] Courtney C. Dutta S. Li Y. (2017). Resolving information asymmetry: Signaling, endorsement, and crowdfunding success. Entrep. Theory Pract. 41, 265–290. doi: 10.1111/etap.12267

[ref22] Daly P. Davy D. (2016). Structural, linguistic and rhetorical features of the entrepreneurial pitch: lessons from dragons’ Den. J. Manag. Dev. 35, 120–132. doi: 10.1108/JMD-05-2014-0049

[ref23] Drover W. Wood M. S. Payne G. T. (2014). The effects of perceived control on venture capitalist investment decisions: A configurational perspective. Entrep. Theory Pract. 38, 833–861. doi: 10.1111/etap.12012

[ref24] Dyer J. H. Singh H. (1998). The relational view: cooperative strategy and sources of interorganizational competitive advantage. Acad. Manag. Rev. 23, 660–679. doi: 10.2307/259056

[ref25] Eddleston K. A. Ladge J. J. Mitteness C. Balachandra L. (2016). Do you see what I see? Signaling effects of gender and firm characteristics on financing entrepreneurial ventures. Entrep. Theory Pract. 40, 489–514. doi: 10.1111/etap.12117

[ref26] Elsbach K. D. Kramer R. M. (2003). Assessing creativity in Hollywood pitch meetings: evidence for a dual-process model of creativity judgments. Acad. Manag. J. 46, 283–301. doi: 10.5465/30040623

[ref27] Harrison R. T. Mason C. Smith D. (2015). Heuristics, learning and the business angel investment decision-making process. Entrepreneurship Reg. Dev. 27, 527–554. doi: 10.1080/08985626.2015.1066875

[ref28] Hatfield E. Cacioppo J. T. Rapson R. L. (1993). Emotional contagion. Curr. Dir. Psychol. Sci. 2, 96–100. doi: 10.1111/1467-8721.ep10770953

[ref29] Hill R. C. Levenhagen M. (1995). Metaphors and mental models: Sensemaking and sensegiving in innovative and entrepreneurial activities. J. Manag. 21, 1057–1074. doi: 10.1177/014920639502100603

[ref30] Ho V. T. Pollack J. M. (2014). Passion isn't always a good thing: examining entrepreneurs’ network centrality and financial performance with a dualistic model of passion. J. Manag. Stud. 51, 433–459. doi: 10.1111/joms.12062

[ref31] Hoppner J. J. Griffith D. A. (2011). The role of reciprocity in clarifying the performance payoff of relational behavior. J. Mark. Res. 48, 920–928. doi: 10.1509/jmkr.48.5.920

[ref32] Huang L. Knight A. P. (2017). Resources and relationships in entrepreneurship: An exchange theory of the development and effects of the entrepreneur-investor relationship. Acad. Manag. Rev. 42, 80–102. doi: 10.5465/amr.2014.0397

[ref33] Hubner S. Baum M. Frese M. (2020). Contagion of entrepreneurial passion: effects on employee outcomes. Entrep. Theory Pract. 44, 1112–1140. doi: 10.1177/1042258719883995

[ref34] Jeng L. A. Wells P. C. (2000). The determinants of venture capital funding: evidence across countries. J. Corp. Finan. 6, 241–289. doi: 10.1016/S0929-1199(00)00003-1

[ref35] Kale P. Singh H. Perlmutter H. (2000). Learning and protection of proprietary assets in strategic alliances: building relational capital. Strateg. Manag. J. 21, 217–237. doi: 10.1002/(SICI)1097-0266(200003)21:3<217::AID-SMJ95>3.0.CO;2-Y

[ref36] Kaufman P. Jayachandran S. Rose R. L. (2006). The role of relational embeddedness in retail buyers' selection of new products. J. Mark. Res. 43, 580–587. doi: 10.1509/jmkr.43.4.580

[ref37] Kirsch D. Goldfarb B. Gera A. (2009). Form or substance: the role of business plans in venture capital decision making. Strateg. Manag. J. 30, 487–515. doi: 10.1002/smj.751

[ref38] Ko E. J. McKelvie A. (2018). Signaling for more money: The roles of founders' human capital and investor prominence in resource acquisition across different stages of firm development. J. Bus. Ventur. 33, 438–454. doi: 10.1016/j.jbusvent.2018.03.001

[ref39] Kumar N. Hibbard J. D. Stern L. W. (1994). The nature and consequences of marketing channel intermediary commitment. Report-Marketing Science Institute Cambridge Massachusetts.

[ref40] Liao J. Welsch H. (2005). Roles of social capital in venture creation: key dimensions and research implications. J. Small Bus. Manag. 43, 345–362. doi: 10.1111/j.1540-627X.2005.00141.x

[ref41] Linder C. Sperber S. (2020). “Mirror, Mirror, on the wall–who is the greatest investor of all?” effects of better-than-average beliefs on venture funding. Eur. Manag. Rev. 17, 407–426. doi: 10.1111/emre.12363

[ref42] Löher J. (2017). The interaction of equity crowdfunding platforms and ventures: an analysis of the preselection process. Ventur. Cap. 19, 51–74. doi: 10.1080/13691066.2016.1252510

[ref44] Maxwell A. L. Lévesque M. (2014). Trustworthiness: A critical ingredient for entrepreneurs seeking investors. Entrep. Theory Pract. 38, 1057–1080. doi: 10.1111/j.1540-6520.2011.00475.x

[ref003] McAllister D. J. (1995). Affect-and cognition-based trust as foundations for interpersonal cooperation in organizations. Acad. Manag. J. 38, 24–59. doi: 10.5465/256727

[ref45] Mitteness C. Sudek R. Cardon M. S. (2012). Angel investor characteristics that determine whether perceived passion leads to higher evaluations of funding potential. J. Bus. Ventur. 27, 592–606. doi: 10.1016/j.jbusvent.2011.11.003

[ref46] Murnieks C. Y. Cardon M. S. Sudek R. White T. D. Brooks W. T. (2016). Drawn to the fire: The role of passion, tenacity and inspirational leadership in angel investing. J. Bus. Ventur. 31, 468–484. doi: 10.1016/j.jbusvent.2016.05.002

[ref47] Nagy B. G. Pollack J. M. Rutherford M. W. Lohrke F. T. (2012). The influence of entrepreneurs’ credentials and impression management behaviors on perceptions of new venture legitimacy. Entrep. Theory Pract. 36, 941–965. doi: 10.1111/j.1540-6520.2012.00539.x

[ref48] Nahapiet J. Ghoshal S. (1998). Social capital, intellectual capital, and the organizational advantage. Acad. Manag. Rev. 23, 242–266. doi: 10.2307/259373

[ref49] O’Connor E. (2002). Storied business: typology, intertextuality, and traffic in entrepreneurial narrative. Int. J. Bus. Commun. 39, 36–54. doi: 10.1177/002194360203900103

[ref50] Ohanian R. (1990). Construction and validation of a scale to measure celebrity endorsers’ perceived expertise, trustworthiness, and attractiveness. Int. J. Advert. 19, 39–52. doi: 10.1080/00913367.1990.10673191

[ref51] Ostgaard T. A. Birley S. (1996). New venture growth and personal networks. J. Bus. Res. 36, 37–50. doi: 10.1016/0148-2963(95)00161-1

[ref005] Palmatier R. W. (2008). Interfirm relational drivers of customer value. J. Mark. 72, 76–89. doi: 10.1509/jmkg.72.4.076

[ref52] Parhankangas A. Renko M. (2017). Linguistic style and crowdfunding success among social and commercial entrepreneurs. J. Bus. Ventur. 32, 215–236. doi: 10.1016/j.jbusvent.2016.11.001

[ref001] Plummer L. A. Allison T. H. Connelly B. L. (2016). Better together? Signaling interactions in new venture pursuit of initial external capital. Acad. Manag. J. 59, 1585–1604. doi: 10.5465/amj.2013.0100

[ref53] Rucker D. D. Petty R. E. (2006). Increasing the effectiveness of communications to consumers: recommendations based on elaboration likelihood and attitude certainty perspectives. J. Public Policy Mark. 25, 39–52. doi: 10.1509/jppm.25.1.39

[ref54] Sahlman W. A. (1997). How to write a great business plan. Harv. Bus. Rev. 75, 98–108. PMID: 10168340

[ref55] Sanchez-Ruiz P. Wood M. S. Long-Ruboyianes A. (2021). Persuasive or polarizing? The influence of entrepreneurs’ use of ingratiation rhetoric on investor funding decisions. J. Bus. Ventur. 36:106120. doi: 10.1016/j.jbusvent.2021.106120

[ref56] Shane S. Drover W. Clingingsmith D. Cerf M. (2020). Founder passion, neural engagement and informal investor interest in startup pitches: An fMRI study. J. Bus. Ventur. 35:105949. doi: 10.1016/j.jbusvent.2019.105949

[ref57] Shane S. Locke E. A. Collins C. J. (2003). Entrepreneurial motivation. Hum. Resour. Manag. Rev. 13, 257–279. doi: 10.1016/S1053-4822(03)00017-2

[ref58] Smith D. A. Lohrke F. T. (2008). Entrepreneurial network development: trusting in the process. J. Bus. Ventur. 61, 315–322. doi: 10.1016/j.jbusres.2007.06.018

[ref59] Spence M. (1974). Market Signaling: Informational Transfer in Hiring and Related Screening Processes (No. 143). Cambridge, Mass: Harvard university press.

[ref60] Spence M. (2002). Signaling in retrospect and the informational structure of markets. Am. Econ. Rev. 92, 434–459. doi: 10.1257/00028280260136200

[ref61] Suvittawat A. (2019). Passions and enthusiasm of small and medium enterprises (SMEs): A case study of Nakorn Ratchasima province, Thailand. Entrep. Sustain.Issues. 6, 1369–1379. doi: 10.9770/jesi.2019.6.3(22)

[ref62] Swap W. Leonard D. Shields M. Abrams L. (2001). Using mentoring and storytelling to transfer knowledge in the workplace. J. Manag. Inf. Syst. 18, 95–114. doi: 10.1080/07421222.2001.11045668

[ref63] Tian X. Wang T. Y. (2014). Tolerance for failure and corporate innovation. Rev. Financ. Stud. 27, 211–255. doi: 10.2139/ssrn.1399707

[ref64] Vallerand R. J. Blanchard C. Mageau G. A. Koestner R. Ratelle C. Léonard M.. (2003). Les passions de l’ame: on obsessive and harmonious passion. J. Pers. Soc. Psychol. 85, 756–767. doi: 10.1037/0022-3514.85.4.756, PMID: 14561128

[ref65] Vismara S. (2018). “Signaling to overcome inefficiencies in Crowdfunding markets,” in The Economics of Crowdfunding. eds. Cumming D. Hornuf L. (Cham: Palgrave Macmillan) doi: 10.1007/978-3-319-66119-3_3

[ref002] Wang Y. (2016). Bringing the Stages Back in: Social Network Ties and Start‐up firms’ Access to Venture Capital in China. Strateg. Entrep. J. 10, 300–317. doi: 10.1002/sej.1229

[ref66] Yang H. Zhang L. Wu Y. Shi H. Xie S. (2021). Influence of entrepreneurial orientation on venture Capitalists’ initial trust. Front. Psychol. 12:633771. doi: 10.3389/fpsyg.2021.633771, PMID: 33868098 PMC8047099

[ref67] Zacharakis A. L. Shepherd D. A. (2001). The nature of information and overconfidence on venture capitalists’ decision making. J. Bus. Ventur. 16, 311–332. doi: 10.1016/S0883-9026(99)00052-X

[ref68] Zhong R. I. (2018). Transparency and firm innovation. J. Account. Econ. 66, 67–93. doi: 10.1016/j.jacceco.2018.02.001

